# Case Report: Post-traumatic *Aeromonas* infection causing severe lower limb soft tissue infection—a report of two surviving cases

**DOI:** 10.3389/fmed.2026.1772258

**Published:** 2026-04-10

**Authors:** Cui Shang, Yushan Zhang, Shili Zhong, Zhengbin Wu, Fuxia Jian

**Affiliations:** Department of Critical Care Medicine, Daping Hospital, Army Medical University, Chongqing, China

**Keywords:** *Aeromonas*, antibiotic therapy, lower limb infection, surgical intervention, traumatic injury

## Abstract

We report two cases of severe lower limb soft tissue infection caused by *Aeromonas* following traumatic injury. A 26-year-old male sustained propeller injury with fractures and developed progressive infection complicated by septic shock and necrotizing fasciitis, requiring left femoral mid-shaft amputation. A 58-year-old male suffered an open tibial plateau fracture and presented with wound infection that resolved after antimicrobial treatment but was followed by tetanus. Both patients had positive cultures for *Aeromonas hydrophila* (and *Aeromonas caviae* in the second case) identified via matrix-assisted laser desorption/ionization time-of-flight mass spectrometry (MALDI-TOF MS) combined with biochemical characterization. Antimicrobial susceptibility testing was performed using the broth microdilution method per CLSI 2024 guidelines, guiding targeted antibiotic therapy. Both patients achieved favorable outcomes after comprehensive management including surgical debridement, targeted antimicrobial treatment, and supportive care, with no recurrence during follow-up. This report summarizes the clinical diagnosis, treatment strategies, and prognosis of post-traumatic *Aeromonas* infection, highlighting the importance of timely microbiological identification, susceptibility testing, and multidisciplinary intervention for improving outcomes in such cases.

## 1 Introduction

*Aeromonas* is a genus of Gram-negative bacilli that predominantly inhabits various aquatic environments and is widely distributed in animals and plants (1). Human infections are mostly caused by contact with contaminated water sources, animal bites, ingestion of contaminated food, or traumatic wound contamination (2, 3). Clinical manifestations in infected patients can involve multiple organ systems, including the eyes, respiratory tract, gastrointestinal tract, skin, limbs, and urinary tract. Severe harm is more likely in individuals with compromised immunity, such as those with liver and kidney failure, malignant tumors, diabetes, and acquired immune deficiencies, often leading to severe sepsis and necrotizing fasciitis (4). A retrospective study in Mexico found a 14.6% mortality rate from bloodstream infections caused by *Aeromonas* in 75 cancer patients (5). Previous studies have shown that the attributable mortality rate of *Aeromonas*-induced sepsis is as high as 33% (3).

Although *Aeromonas* infections are rare and opportunistic, their early clinical manifestations are non-specific, leading to difficulties in timely and accurate diagnosis. This paper presents two cases of severe post-traumatic lower limb infection caused by *Aeromonas*, one of which was complicated with lower limb gangrene requiring amputation, and the other with tetanus after initial infection control. Both patients survived, and this study summarizes the clinical diagnosis and treatment experience of such cases to provide a reference for the clinical management of post-traumatic *Aeromonas* infection.

## 2 Case description

### 2.1 Case 1

A 26-year-old male with no underlying chronic diseases suffered a propeller injury after falling into water during sailing and was admitted to a local hospital with hemorrhagic shock. Initial examinations revealed a left femoral shaft fracture, bilateral lateral tibial plateau fractures, and a right fibular head fracture. The patient received empirical antimicrobial treatment with ceftriaxone + metronidazole, but fever persisted and infection progressed. He was transferred to our hospital on the 4th day after injury.

**Figure 1:**
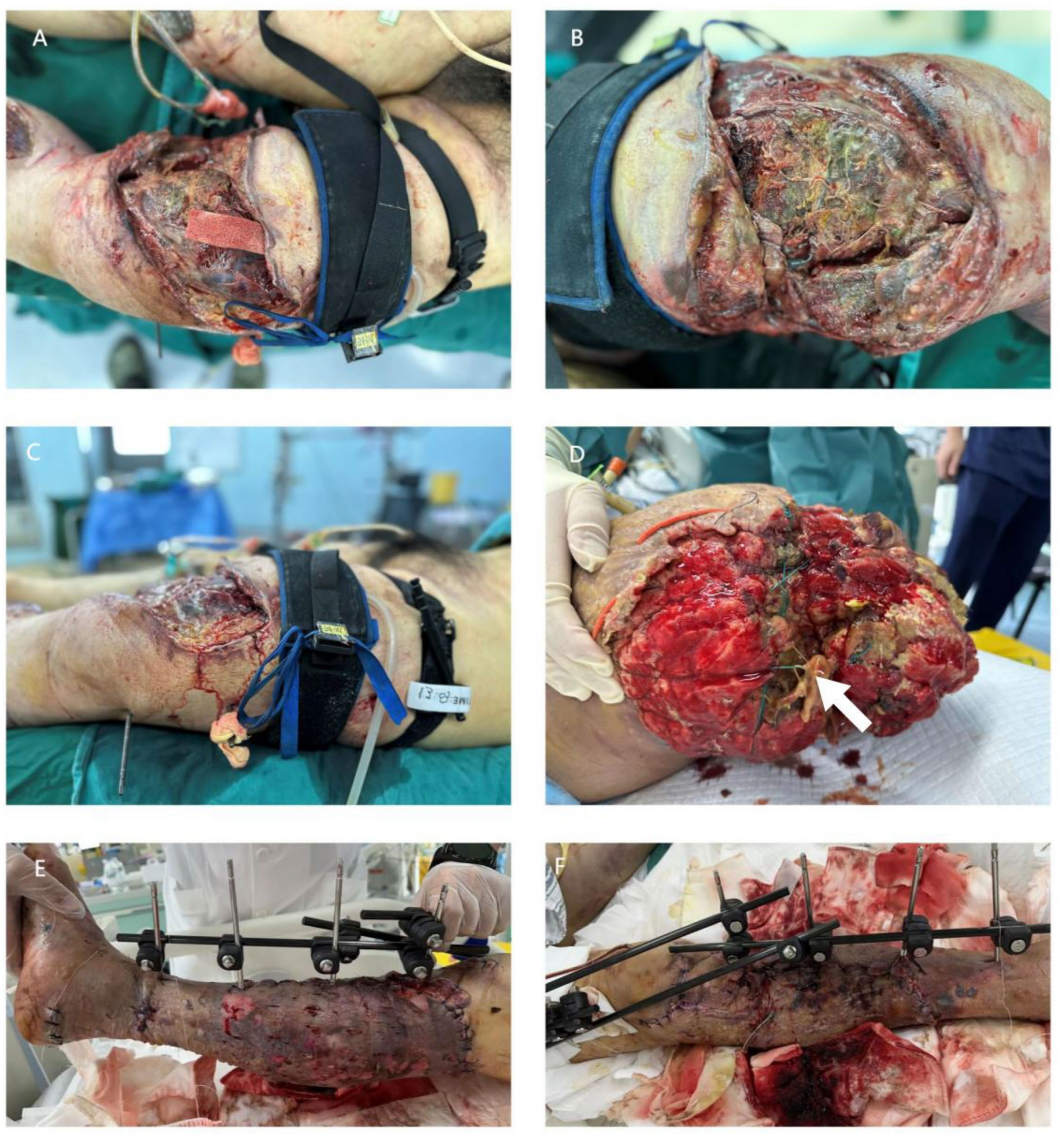
Wound status of two patients. Case 1: **(A–D)** Wound status at admission and during surgery. **(A)** Left lower limb (anterolateral aspect of the thigh) on admission, showing extensive swelling and subcutaneous emphysema (the main difference from **C** is that this figure shows the external clinical manifestation of the wound at admission, while **C** shows the intra-operative microscopic view of extensive necrotic muscle tissue in the deep layer of the left thigh); **(B)** Left thigh wound (medial aspect) with bloody foul-smelling secretions; **(C)** intraoperative view of left thigh (anterior compartment) with extensive necrotic muscle tissue and gray-white moss-like necrotic tissue; **(D)** left thigh wound (deep muscle layer) with visible foreign body fragments (rust, sawdust) (white arrow pointing to foreign body fragments). Case 2: **(E,F)** Wound status at admission to the local hospital. **(E)** Left knee (tibial plateau area) with open tibial plateau fracture and obvious wound contamination with soil and gravel; **(F)** Left tibial plateau wound after surgery, with purulent secretions mixed with necrotic tissue exuding from the wound edge.

On admission to our hospital, physical examination showed extensive swelling and subcutaneous emphysema of both lower limbs, bloody foul-smelling secretions from the wound, and non-palpable left dorsalis pedis artery pulse (Figures 1A–D). Emergency CTA showed occlusion of the left femoral artery and distal vessels. Emergency surgery included debridement, necrotic tissue resection, VSD placement, and left femoral artery exploration. Intraoperatively, extensive necrotic muscle and foreign body fragments (rust, sawdust) were found and completely debrided. Meanwhile, imipenem 1.0 g was administered intravenously every 8 h for antimicrobial treatment.

On the 3rd day after surgery, the patient developed sudden hypotension (81/50 mmHg) and hemoglobin dropped to 62 g/L, with dark red bleeding from the left lower limb wound. Emergency left femoral mid-shaft amputation was performed. Postoperatively, persistent fever continued.

#### 2.1.1 Propeller sampling, culture, and contamination exclusion

Propeller fragments were immediately placed into a sterile sealed container to avoid environmental contamination. Prior to culture, the fragments were rinsed three times with sterile normal saline to remove non-adherent surface contaminants, then soaked in sterile normal saline for 30 min and vortexed for 10 min to elute adherent bacteria. The supernatant was inoculated onto blood agar and MacConkey agar and incubated aerobically at 37 °C for 24–48 h. Blank control cultures were prepared using the sterile saline and culture medium. No bacterial growth was observed in the control groups, thus excluding experimental contamination.

Blood culture, wound secretion culture, and propeller debris culture all grew *A. hydrophila*. Identification was performed using MALDI-TOF MS combined with the VITEK 2 system.

#### 2.1.2 Susceptibility profile and testing method

Antimicrobial susceptibility testing was performed using the broth microdilution method according to the Clinical and Laboratory Standards Institute (CLSI) 2024 guidelines. The *A. hydrophila* isolate was susceptible to imipenem, meropenem, levofloxacin, and piperacillin tazobactam, but resistant to ampicillin and cefazolin.

Targeted antimicrobial therapy was initiated with imipenem 1.0 g ivgtt q8h, teicoplanin 0.4 g ivgtt qd, and levofloxacin 0.5 g ivgtt qd.

#### 2.1.3 Control cultures and clinical improvement criteria

During targeted antimicrobial treatment, control cultures (blood and wound secretions) were performed every 3 days. All cultures became negative for *A. hydrophila* from day 7 of treatment onward. Clinical improvement criteria were defined as follows: (1) Fever resolution (temperature < 37.5 °C for ≥ 72 h); (2) Normalization of white blood cell count and C-reactive protein; (3) No obvious purulent or bloody exudate from the wound; (4) Visible growth of fresh granulation tissue.

The infection was well controlled on day 14 of treatment. The patient subsequently underwent 7 additional debridements and autologous skin grafting and was cured after 3 months of comprehensive treatment.

### 2.2 Case 2

**Figure 2:**
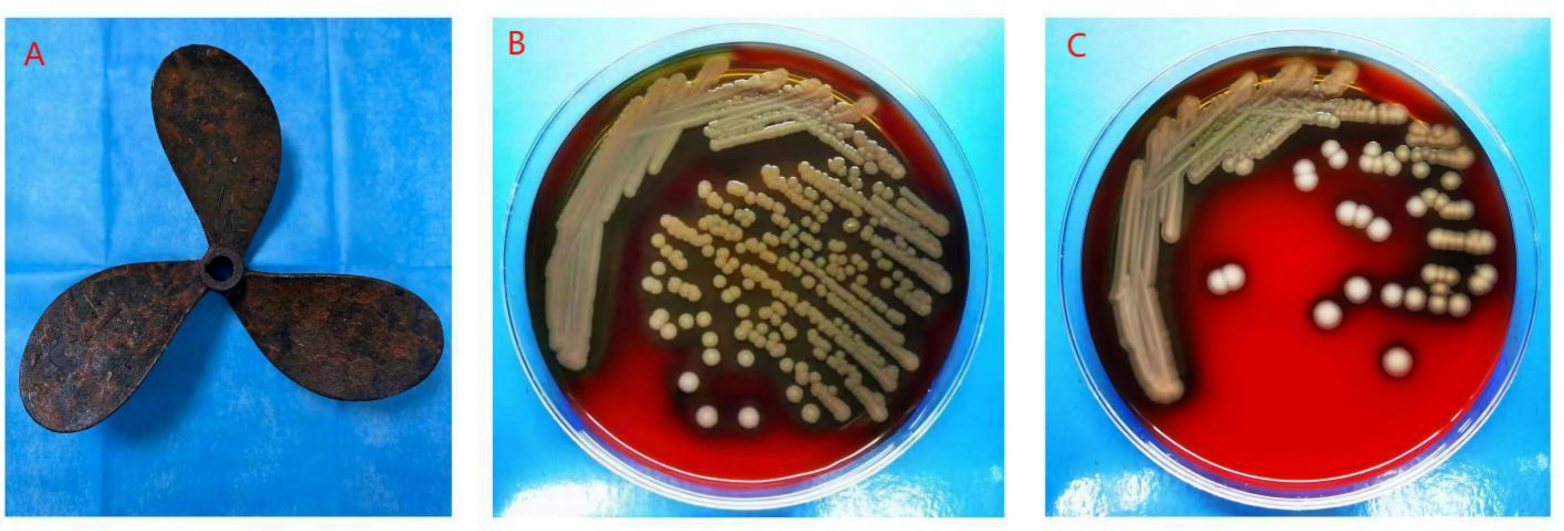
Propeller and wound culture. **(A)** Culture result of propeller debris (Gram staining, ×1,000), showing pure growth of *Aeromonas hydrophila* (Gram-negative bacilli, short rod-shaped, single polar flagellum); **(B)** Culture result of left lower limb wound secretion (blood agar culture, 37 °C, 24 h), showing heavy growth of *Aeromonas hydrophila* (round, moist, pale yellow colonies); **(C)** Blood culture result (BACTEC FX blood culture system + blood agar culture), showing positive for *Aeromonas hydrophila* (no other pathogenic bacteria were isolated).

A 58-year-old male with no underlying chronic diseases suffered an open left tibial plateau fracture in a car accident, and was treated in a local hospital with emergency debridement, antimicrobial treatment, tetanus toxoid vaccination and external fixation of the fractures (Figures 1E,F). Postoperatively, the patient had recurrent fever, and the wound produced purulent secretions mixed with necrotic tissue. Wound secretion samples were inoculated on blood agar and MacConkey agar for aerobic culture at 37 °C for 24–48 h; taxonomic identification was completed by MALDI-TOF MS combined with biochemical characterization, and drug sensitivity testing was performed by broth microdilution method according to CLSI 2024 guidelines (6); wound secretion culture was positive for *A. hydrophila* and *A. caviae* (susceptibility profile: susceptible to piperacillin-tazobactam, levofloxacin, imipenem, resistant to ampicillin, cefuroxime) (Figure 2); blood culture was negative. Due to poor infection control, the patient was transferred to our hospital.

After admission, the antimicrobial regimen was adjusted based on the results of antimicrobial susceptibility testing, and piperacillin-tazobactam 4.5 g q6h was intravenously administered for a 7-day course of antimicrobial therapy. Repeated wound secretion culture was performed during the treatment, and the results turned negative for *Aeromonas* after 5 days of treatment; the patient received 5 times of wound debridement and autologous skin grafting, and the infection was controlled with no obvious fever or wound exudation, and was discharged from the hospital after wound healing. One month after discharge, the patient developed trismus, dyspnea and opisthotonos, and was definitely diagnosed with tetanus after admission to our hospital. He was transferred to the intensive care unit (ICU) for tracheotomy and invasive mechanical ventilation support. After 3 months of anti-tetanus, respiratory support and symptomatic treatment, the patient’s neuromuscular symptoms completely relieved, respiratory function recovered, and he was discharged with no residual neurological dysfunction.

## 3 Discussion and conclusions

### 3.1 Clinical characteristics and pathogenic factors of post-traumatic *Aeromonas* infection

*Aeromonas* is a common Gram-negative bacterium found in nature but is rare in human infections. First identified in feces in 1961, it primarily causes gastroenteritis, wound infections, and septicemia (7). The mortality rate associated with *Aeromonas* septicemia is 33%, with common symptoms including fever, abdominal pain, septic shock, and dyspnea (3). Wound infections caused by *Aeromonas* can progress to severe necrotizing fasciitis, especially in individuals with severe liver disease and malignant tumors, where the mortality rate can reach 60–75% (3). Clinically, 95% of pathogenic *Aeromonas* strains are *Aeromonas caviae*, *Aeromonas* dhakensis, *Aeromonas veronii* and *A. hydrophila* (7), which were also the main pathogenic bacteria in the two cases reported in this study. The main clinical features of the two patients were traumatic wound infection, progressive local tissue necrosis, and systemic inflammatory response, and one patient developed bacteremia, which is consistent with the clinical characteristics of severe *Aeromonas* infection reported in the literature.

**Figure 3:**
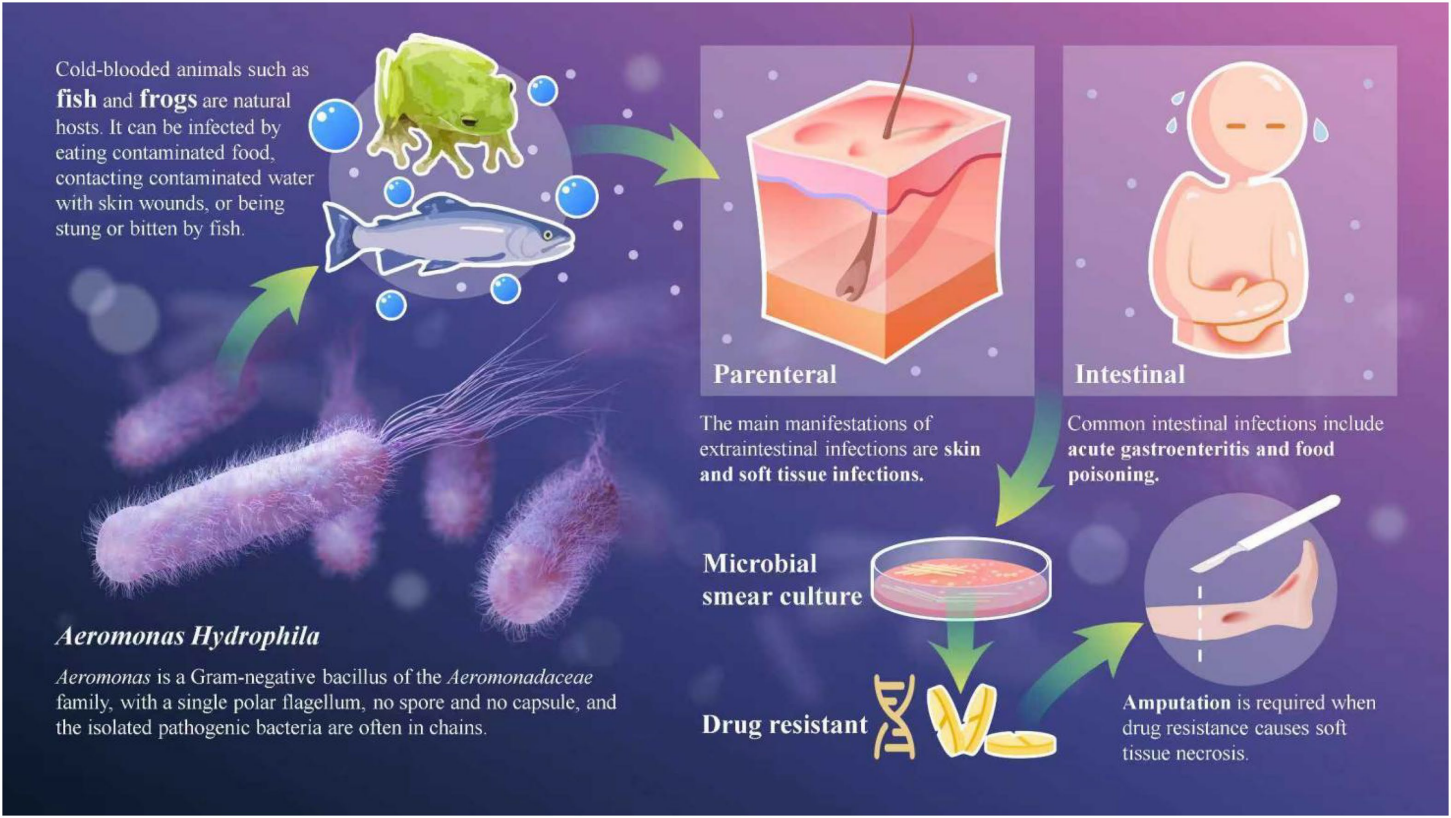
Pathogenesis and clinical manifestations of *Aeromonas. Aeromonas* is a Gram-negative bacillus of the *Aeromonadaceae* family, with a single polar flagellum, no spore and no capsule, and the isolated pathogenic bacteria are often in chains. Cold-blooded animals such as fish and frogs are natural hosts of *Aeromonas*. Human infection mainly occurs through two routes: parenteral (contact of traumatic wounds with contaminated water, or bite/sting by aquatic animals) and intestinal (ingestion of contaminated aquatic products). The main clinical manifestations include intestinal infections (acute gastroenteritis, food poisoning) and extraintestinal infections (skin and soft tissue infections, sepsis, necrotizing fasciitis, etc.). Microbial smear and culture are the gold standard for diagnosis. For severe infections with drug resistance-induced soft tissue necrosis and gangrene, surgical amputation is required to save the patient’s life.

*Aeromonas* infection is typically opportunistic, occurring when skin and mucous membranes are compromised due to direct exposure to fresh water, salt water, lakes, sewage ditches, or animal attacks by snakes, bears, and other animals (8, 9). Infections can also result from iatrogenic sources, such as damaged skin flaps from leech treatment or the entry of *A. hydrophila* into the bloodstream during hemodialysis (10, 11). In addition, ingestion of raw or undercooked contaminated aquatic products (fish, oysters, mussels) is a common cause of intestinal *Aeromonas* infection (7, 12) (Figure 3). Immunocompromised individuals (diabetes, cirrhosis, end-stage renal disease, organ transplantation) are at high risk of severe *Aeromonas* infection and septic shock (Figure 3) (13). Notably, the two patients in this report had no underlying chronic diseases or immune deficiency, but traumatic injury led to severe skin and soft tissue damage and loss of barrier function, which became the main predisposing factor for *Aeromonas* infection. This suggests that traumatic wound contamination is an important cause of *Aeromonas* infection in immunocompetent individuals, and clinicians should pay attention to this potential infection risk in the treatment of traumatic patients.

### 3.2 Diagnosis and treatment of post-traumatic *Aeromonas* infection

The early clinical manifestations of *Aeromonas* infection are non-specific, and timely diagnosis is mainly based on microbial culture and identification (3). Accurate identification of *Aeromonas* species is a major clinical challenge, as traditional biochemical characterization has low resolution for closely related species; 16S rRNA gene sequencing, a commonly used molecular method, also has limitations in distinguishing between some *Aeromonas* species due to high sequence homology (14). MALDI-TOF MS has become a rapid and accurate method for the identification of *Aeromonas* in clinical laboratories (15), and whole genome sequencing (WGS) is the gold standard for species differentiation and phylogenetic analysis of *Aeromonas*, which can accurately distinguish between different species and detect virulence and resistance genes (7).

For traumatic wound infection patients with persistent fever, progressive local tissue necrosis and poor response to empirical antibiotic therapy, *Aeromonas* infection should be suspected, and blood culture and wound secretion culture should be performed as early as possible to clarify the pathogenic bacteria. At the same time, drug sensitivity testing should be completed to guide the selection of targeted antibiotics, which is the key to improving the treatment success rate. In this study, Case 1 was diagnosed with *Aeromonas* bacteremia by positive blood and wound secretion culture, and Case 2 was diagnosed by positive wound secretion culture; the pathogenic bacteria were identified as *A. hydrophila* and/or *A. caviae* by microbial identification, which provided a reliable basis for subsequent targeted antibiotic therapy.

*Aeromonas* resistance is a major challenge in clinical treatment. *Aeromonas* can produce *β*-lactamase, metallo-β-lactamase and carbapenemase, which can hydrolyze penicillins, cephalosporins and carbapenems, leading to drug resistance (16). In addition, horizontal transfer of resistance genes between strains and irrational use of antibiotics further exacerbate bacterial drug resistance (16). Transcriptomic studies have shown that *A. hydrophila* can activate a variety of virulence factors (lipase, toxin, protease) after infection, leading to extensive damage to host tissue and organ functions (17). Fortunately, the pathogenic bacteria isolated in these two cases were susceptible to multiple clinically available antimicrobial agents based on susceptibility testing, and targeted antimicrobial therapy yielded favorable therapeutic effects—an important factor contributing to the successful management of both patients. For clinical *Aeromonas* infection, empirical broad-spectrum antibiotics can be used initially according to the condition, and the antibiotic regimen should be adjusted in time based on drug sensitivity test results to avoid the use of ineffective antibiotics and improve the treatment effect.

### 3.3 Analysis of neuromuscular complications in case 2

*Aeromonas* infection is often caused by trauma, increasing the risk of concurrent bacterial infections, especially *Clostridium tetani* (18). This bacterium can cause respiratory paralysis due to uncontrolled muscle spasms, resulting in a high mortality rate (19). Case 2 presented with trismus, dyspnea and opisthotonos 1 month after discharge from the hospital due to wound healing, which are typical clinical manifestations of tetanus. The patient had a history of open tibial plateau fracture with obvious wound contamination by soil and gravel, and although tetanus toxoid was vaccinated after injury, the incomplete debridement of the wound may have led to residual *Clostridium tetani* spores, which germinated and produced tetanus toxin in the anaerobic microenvironment of the wound, eventually inducing tetanus. An emergency mid-femur amputation was performed. Concurrent empiric antimicrobial treatment therapy, such as combining broad-spectrum beta-lactamase antibiotics with aminoglycosides, is also recommended (18, 20, 21).

The effects of *Aeromonas* on the human nervous system are rarely reported. Previous studies have only found that *Aeromonas* infection can adversely affect the nervous system of fish: the extracellular product acetylcholinesterase of *A. hydrophila* can cause movement imbalances, weakened respiration, and fish death (22), and fish infected with *Aeromonas* may exhibit stress-related excessive movement, which may be related to changes in the hypothalamic–pituitary-adrenocortical axis and increased expression and release of hspa12a and crh in the brain (23). However, these findings are limited to aquatic animals and cannot be extrapolated to humans, and there is no direct clinical or experimental evidence that *Aeromonas* can cause neuromuscular damage in humans.

However, this study has a limitation that anaerobic culture of the wound secretion was not performed, and no direct microbiological evidence of *Clostridium tetani* infection was obtained; in addition, the specific anti-tetanus treatment measures (e.g., use of tetanus immunoglobulin, muscle relaxants) were not explicitly recorded in the clinical data. Although the patient received tetanus vaccine injection after injury, the occurrence of tetanus may be related to incomplete wound debridement leading to residual *Clostridium tetani* spores, which germinated and produced toxins in an anaerobic environment. Therefore, the neuromuscular complications in Case 2 are definitely caused by tetanus, and there is no direct correlation with *Aeromonas* infection.

### 3.4 Clinical implications and limitations

The two cases of post-traumatic *Aeromonas*-induced severe lower limb infection reported in this study have important clinical implications: first, for traumatic patients with wound contamination (especially aquatic environment contamination), clinicians should be alert to the risk of *Aeromonas* infection, and perform microbial culture (including blood culture and wound secretion culture) in a timely manner for patients with persistent fever and poor wound healing to clarify the pathogenic bacteria; second, the treatment of severe *Aeromonas* infection should adhere to the combination of antibiotics and surgery, with targeted antibiotic therapy based on drug sensitivity testing and timely surgical debridement or amputation according to the condition; third, in the treatment of traumatic patients, comprehensive preventive measures should be taken to reduce the risk of secondary infections such as tetanus, including thorough wound debridement and standardized immunoprophylaxis.

This study has certain limitations: it is a single-center case report with a small number of cases, and lacks large-sample clinical data support; the follow-up time of the two patients is relatively short, and the long-term prognosis needs to be further observed; the pathogenic mechanism of *Aeromonas* in traumatic wound infection and the interaction between *Aeromonas* and other bacteria need to be further studied by basic experiments; in addition, anaerobic culture was not performed for Case 2, and no direct microbiological evidence of *Clostridium tetani* infection was obtained, which is a limitation of the diagnosis of tetanus in this case.

## Data Availability

The original contributions presented in the study are included in the article/supplementary material, further inquiries can be directed to the corresponding authors.
